# Metabolic Mechanisms of Hexavalent Chromium-Induced Splenic Immune Injury via Oxidative Stress and Ferroptosis Pathways in New Zealand Rabbits

**DOI:** 10.3390/metabo16060430

**Published:** 2026-06-18

**Authors:** Junzhao Yuan, Jiaqi Zhang, Jinxing Song, Lingling Liu, Hang Liu, Shuangxing Jin, Xiaoli Ren

**Affiliations:** 1 Zhengzhou Key Laboratory of Animal Nutrition Metabolic and Poisoning Diseases, College of Veterinary Medicine, Henan University of Animal Husbandry and Economy, Zhengzhou 450046, China; 2 College of Veterinary Medicine, Henan University of Animal Husbandry and Economy, Zhengzhou 450046, China

**Keywords:** hexavalent chromium, rabbit spleen, immunotoxicity, redox metabolism, ferroptosis

## Abstract

**Background**: Hexavalent chromium (Cr(VI)) is a widespread environmental toxic heavy metal with strong oxidative properties; however, its immunotoxicity and metabolic mechanisms in rabbit spleen remain largely unclear. **Methods**: In this study, New Zealand rabbits were exposed to 0, 12.5, 25, and 50 mg/L Cr(VI) (as potassium dichromate, K_2_Cr_2_O_7_) via drinking water for four weeks to investigate splenic damage and the underlying molecular pathways. Spleen pathological injury was evaluated by hematoxylin and eosin (H&E) staining, and the distribution of T cells, B cells, and macrophages was assessed by immunohistochemistry. Antioxidant enzyme activities and antioxidant substance levels were determined using ELISA, and the relative mRNA expression of immune factor genes, antioxidant-related genes, and ferroptosis-related genes was quantified by quantitative real-time PCR (qRT-PCR). In addition, the distribution of iron in splenic tissue was detected by enhanced Prussian blue staining. **Results**: Our results demonstrate that high-dose Cr(VI) significantly inhibited body weight gain, induced lymphocyte atrophy, vacuolization, and widening of intercellular spaces in the splenic white pulp. Furthermore, Cr(VI) reduced T and B lymphocyte populations, promoted macrophage infiltration and inflammatory cytokine gene expression in a concentration-dependent manner, impaired total antioxidant capacity, and led to a decrease in glutathione (GSH) levels in the spleen. Additionally, Cr(VI) exposure increased iron accumulation, activated the ACSL4–NOX lipid peroxidation cascade, and downregulated *GPX4* expression, ultimately triggering ferroptosis. **Conclusions**: These findings reveal that Cr(VI) causes splenic immune injury by disrupting oxidative homeostasis and inducing ferroptosis, providing novel insights for evaluating immunotoxicity and identifying metabolic targets under Cr(VI) pollution.

## 1. Introduction

Chromium (Cr) naturally occurs mainly in the form of compounds in Cr ores such as ultramafic rocks, basalts, and serpentinites [1]. While metallic Cr is valued for its high melting point, hardness, and electrical conductivity, its ionic forms, particularly Cr(VI), are characterized by strong oxidizing potential. Cr is a widely applied industrial metal in tanning, electroplating, dye synthesis, and textile industries. In aqueous environments, Cr mainly exists in two stable valence states, trivalent chromium (Cr(III)) and hexavalent chromium (Cr(VI)), which exhibit completely different biological properties. As an essential trace element for animals and humans, Cr(III) participates in the regulation of carbohydrate, lipid and protein metabolism, and moderate Cr(III) supplementation can effectively improve animal growth, reproduction and immune performance [2]. In contrast, Cr(VI) is a heavy metal ion that causes environmental pollution, and its toxicity to organisms is approximately 100 to 1000 times that of Cr(III), primarily owing to its strong oxidation characteristics [3]. Unlike Cr(III), Cr(VI) can penetrate cells through non-specific sulfate or phosphate ion channels on the cell membrane. Once inside cells, Cr(VI) gradually consumes reducing molecules such as glutathione (GSH) and ascorbic acid and is reduced to Cr(III); during this process, the intermediate Cr(IV) and Cr(V) species bind to intracellular DNA, damage genetic material, and generate substantial amounts of reactive oxygen species (ROS), leading to cellular injury and even carcinogenesis [4,5].

Cr(VI) exposure occurs via inhalation and dermal contact in industrial settings, causing dermatitis and lung cancer, while dietary ingestion induces acute symptoms such as vomiting and convulsions [6]. Following oral ingestion, gut microbiota can enzymatically reduce Cr(VI) to the less toxic Cr(III); however, reversible oxidation of Cr(III) back to Cr(VI) can also occur under certain intestinal conditions, thereby aggravating biological toxicity risks [7,8,9]. At present, industrial production activities have caused widespread Cr(VI) contamination in global water and soil environments [10,11]. Notably, Cr-containing wastewater and Cr residues discharged from China’s electroplating industry constitute a major source of environmental Cr pollution [12,13,14,15]. Moreover, Cr(III) in natural water sediments and water bodies can be converted into toxic Cr(VI) via chemical oxidation and photo-oxidation reactions under natural conditions, leading to persistent environmental Cr(VI) pollution [16]. Cr(VI) accumulates in animals through drinking water and food chains, highlighting the importance of elucidating its toxic mechanisms.

The toxic mechanism of Cr(VI) involves DNA damage, impaired genomic stability, oxidative stress, and ROS generation [17,18]. After entering the bloodstream, Cr(VI) is rapidly reduced to Cr(III) by red blood cells and plasma components, rendering the accumulation pattern of Cr(III) in the spleen markedly different from that in the liver and kidney [19]. Studies have shown that after oral exposure to Cr(VI), splenic Cr content is lower than that in the liver and kidney but still increases in a dose- and time-dependent manner, with a biological half-life of approximately 3–7 days, and is mainly deposited in red pulp macrophages as Cr(III)–protein complexes [20]. Although studies have confirmed the pathological effects of Cr(VI) on organs such as the kidney, liver, lung, nerves, and testis, investigations into its effects on spleen tissue remain relatively scarce. As the largest immune organ in rabbits, the spleen is richly populated with immune cells and is mainly divided into three compartments: white pulp (WP) consists of dense lymphocytes, including periarterial lymphatic sheaths (PALS) where T cells aggregate and lymphoid follicles dominated by B cells, serving as the core region for adaptive immune responses; red pulp (RP) comprises the vast majority of splenic parenchyma, being interwoven by splenic cords and sinuses, abundant in red blood cells and various leukocytes with innate immune functions; the marginal zone (MZ), located at the junction of the white pulp (WP) and red pulp (RP), exhibits a loose structure rich in macrophages and various lymphocytes, serving as an important channel for blood-borne antigens to enter the spleen and initiate immune responses, as well as a key node for lymphocyte recirculation [21]. High concentrations of Cr accumulation can cause pathological damage to the spleen tissue of SD rats, including WP atrophy, RP congestion, and macrophage activation, thereby leading to immune inhibition and an inflammatory response [22]. Related Cr accumulation toxicity tests in mice showed that splenic Cr content in the high-dose group was significantly higher than that in other tissues, and concomitantly, with increasing poisoning dose, the number of lymphocytes in mice decreased while neutrophils increased, further confirming the inhibitory effect of Cr accumulation on immune function and the induction of an inflammatory response [23]. Splenic Cr residue has been proposed as a sensitive biomarker for chronic exposure [24]. However, the metabolic damage to splenic immune cells, particularly alterations in immune cell distribution, following Cr(VI) exposure in New Zealand rabbits remains poorly characterized.

Cr(VI) in the environment primarily causes oxidative stress-mediated metabolic damage to cells [25]. The main antioxidant enzymes and small-molecule antioxidants in animal cells include superoxide dismutase (SOD), glutathione peroxidase (GSH-Px), glutathione reductase, catalase (CAT), superoxide reductase, and reduced glutathione (GSH) [26,27]; their disruption triggers ferroptosis, an iron-dependent cell death driven by lipid peroxidation and marked by glutathione peroxidase 4 (GPX4, *GPX4*) inactivation and GSH depletion [28]. GPX4 utilizes GSH to detoxify lipid peroxides, and its loss leads to lethal membrane damage. The System Xc^−^/GSH/GPX4 axis is central to ferroptosis defense: Nuclear factor E2-related factor 2 (Nrf2, *NFE2L2*) dissociates from Keap1 under stress and upregulates the System Xc^−^ light chain subunit (xCT/SLC7A11) and GPX4, enhancing GSH synthesis and lipid peroxide clearance [29,30,31,32,33]. Additionally, p53 represses SLC7A11, whereas activating transcription factor 4 (ATF4, *ATF4*) induces xCT, jointly modulating ferroptosis sensitivity [34,35]. Conversely, 15-lipoxygenase-1 (ALOX15, *ALOX15*) drives ferroptosis by catalyzing peroxidation of polyunsaturated fatty acids (PUFAs)-containing phospholipids, directly compromising membrane integrity [27,36]. Acyl-CoA synthetase long-chain family member 4 (ACSL4, *ACSL4*) provides PUFA substrates, and Fe^2+^ enhances ALOX15 activity while synergizing with NOX-derived ROS [37,38,39]. Studies have shown that *ALOX15* gene knockout or pharmacological inhibition can significantly attenuate ferroptotic cell damage induced by Erastin/RSL3, while *ALOX15* overexpression enhances cellular susceptibility to ferroptosis, confirming its core pro-ferroptotic role in the execution stage of ferroptosis [40,41,42]. Prostaglandin-endoperoxide synthase 2 (PTGS2, *PTGS2*), heme oxygenase 1 (HO-1, *HMOX1*), and lactoferrin (LTF, *LTF*) act as critical regulators of ferroptosis. In addition, metal regulatory transcription factor 1 (MTF1, *MTF1*), as well as NADPH oxidase isoforms NOX1, NOX2, and NOX4, are also involved in this progression. Collectively, these genes modulate ferroptosis through multiple regulatory dimensions, including the control of lipid peroxidation metabolism, antioxidant metabolic defense, iron homeostasis, and autophagy-related crosstalk. Although ferroptosis has been studied in splenic cells under hypoxia, infection, and inflammation, its role in Cr(VI)-induced oxidative damage in the rabbit spleen is virtually unexplored [43,44,45].

Cr(VI) pollution is a mounting global challenge that causes bioaccumulation and chronic toxicity in animals and humans [46,47]. Rabbits share metabolic similarities with humans and serve as translational models for heavy metal toxicology [48]; their splenic immune cell distribution resembles that of humans, making them suitable for evaluating the System Xc^−^/GSH/GPX4 axis and ferroptosis. Previous studies have focused on nonspecific oxidative damage by Cr(VI); whether it triggers ferroptosis in the spleen remains unknown. This study is the first to investigate the effects of Cr(VI) exposure on the splenic ferroptosis pathway in New Zealand rabbits, aiming to reveal a novel mechanism of its immunotoxicity.

## 2. Materials and Methods

### 2.1. Experimental Animal Feeding and Grouping

**Figure 1:**
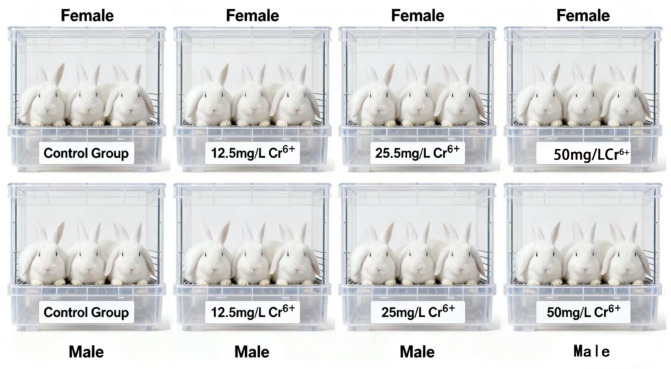
Overview of experimental grouping.

Twenty-four healthy 30-day-old New Zealand white rabbits were purchased from Du Guoli Breeding Cooperative in Yuanyang, Henan Province, and housed in Individually Ventilated Cage (IVC) systems equipped with constant temperature and ventilation in the animal facility. The rabbits were randomly assigned to a control group, a low-concentration exposure group (12.5 mg/L Cr(VI)), a medium-concentration exposure group (25 mg/L Cr(VI)), and a high-concentration exposure group (50 mg/L Cr(VI)), for a total of 4 groups. Each group consisted of two cages, each containing 3 male and 3 female rabbits, i.e., 6 rabbits per group. The experimental design is illustrated in Figure 1. To minimize discomfort to the animals, Cr(VI) was added to drinking water in the form of potassium dichromate (K_2_Cr_2_O_7_), with the concentration expressed as Cr(VI). The Cr(VI) concentrations were determined by reviewing pollution levels in relevant contaminated areas worldwide and referring to Zhao Xiujun’s study, which reported that the Cr(VI) concentration in groundwater near Jinchangbao, Jinzhou, Liaoning Province, China, reached 28.64 mg/L, adjacent to a ferroalloy factory [49]. Additionally, Study showed that the Cr(VI) concentration in groundwater at a contaminated site in Xinxiang, Henan Province, was as high as 10 mg/L [50]. Notably, the maximum Cr(VI) concentration in groundwater in Kanpur, India, has been documented to reach 12.5 mg/L (250 times higher than the WHO permissible limit), highlighting the severity of Cr(VI) contamination in certain regions [51]. Therefore, the Cr(VI) concentration gradient was established after careful consideration. Drinking water containing 0, 12.5, 25, and 50 mg/L Cr(VI), prepared by dissolving appropriate amounts of K_2_Cr_2_O_7_ powder, was provided to the control and three experimental groups, respectively. The New Zealand rabbits were allowed free access to drinking water. New Zealand rabbits were fed a full-price fattening diet twice daily; the exposure period lasted 4 weeks, during which the rabbits had ad libitum access to water. The rabbit facility was cleaned every 3 days, and body weights were recorded simultaneously. This animal experiment was conducted in strict accordance with the Guidelines for the Care and Use of Laboratory Animals issued by the China State Science and Technology Commission and was approved by the Ethics Committee of Henan University of Animal Husbandry and Economy (Protocol No. 202302001).

### 2.2. Materials and Reagents

K_2_Cr_2_O_7_ (analytical grade, 500 g) was purchased from Dene Chemical Reagent Co., Ltd. (Tianjin, China). TRIzol reagent was obtained from Tiangen Biotech Co., Ltd. (Beijing, China). The reverse transcription kit FastKing cDNA First Strand Synthesis Kit and the quantitative PCR reagent FastReal Rapid Fluorescent Quantitative PCR Premix (SYBR Green) were obtained from Zhengzhou Dongge Bioengineering Technology Co., Ltd. (Zhengzhou, China). Neutral tissue fixative (G1101-500ML) was purchased from Sevier Biotechnology Co., Ltd. (Wuhan, China). The Catalase (CAT) Assay Kit (Visible Light, Catalog No. A007-1-1), the Total Antioxidant Capacity (T-AOC) Assay Kit (FRAP method, A015-3-1), the Total Superoxide Dismutase (T-SOD) Assay Kit (Hydroxylamine method, A001-1), and the Reduced Glutathione (GSH) Assay Kit (A006-2-1) were obtained from Nanjing Jiancheng Bioengineering Institute (Nanjing, China). The Enhanced BCA Protein Assay Kit (P0009, 5000 assays) was purchased from Beyotime Biotechnology Co., Ltd. (Shanghai, China).

### 2.3. Sample Collection and Processing

After the experimental period, New Zealand rabbits were anesthetized with Sumianxin II and then euthanized by blood collection. The entire spleen was carefully dissected, rinsed with physiological saline to remove residual blood, and attached fat and other impurities were removed with tissue forceps; surface saline was absorbed with filter paper, after which the spleen weight was recorded. Subsequently, tissue specimens were taken from the splenic parenchyma, cut into approximately 5 mm^3^ tissue blocks, and placed into 1.5 mL EP tubes. A portion of the spleen tissue was snap-frozen in liquid nitrogen for later measurement of antioxidant enzyme activities and antioxidant levels. The spleen tissue preserved in the 1.5 mL EP tube was fixed for pathological sectioning, and the liquid-nitrogen-frozen spleen tissue was used for qRT-PCR analysis of antioxidant genes, ferroptosis genes, and cytokines.

### 2.4. Determination of Spleen Organ Index

Body weight (g) and spleen weight (g) of New Zealand rabbits were accurately measured with an electronic balance, and the data for each rabbit were recorded. The spleen coefficient was calculated according to the formula: spleen coefficient = spleen weight (g)/body weight (g). Group data were compared and analyzed to determine the trend of spleen coefficient changes.

### 2.5. Spleen Pathological Examination

Fresh spleen tissue blocks of approximately 1 cm^3^ were quickly excised from New Zealand rabbits with a scalpel and immediately placed in neutral tissue fixative for overnight fixation. After dehydration, clearing, paraffin infiltration, and embedding, the embedded spleen tissue was sectioned into 4–6 μm thick slices using a microtome. After section picking and baking, the slices were mounted onto glass slides. Following hematoxylin-eosin (H&E) staining, the prepared pathological sections were observed under an inverted microscope (Motic, AE31E, Xiamen, China), and images were captured to document the corresponding pathological changes.

### 2.6. Measurement of Antioxidant Capacity in Spleen Tissue

Spleen tissue samples from each group were removed from liquid nitrogen; 6 samples per group were used. Twenty-milligram spleen tissue specimens from each New Zealand rabbit were taken, mixed with ice-cold PBS at a ratio of 1:9, and minced with surgical scissors in ice-cold PBS to prepare tissue homogenates. The homogenate was divided into two aliquots: one for the measurement of antioxidant parameters and the other for protein concentration determination. T-AOC level (U/mgprot), GSH content (μmol/gprot), CAT and T-SOD activities (U/mgprot) in different groups were measured to elucidate changes in antioxidant enzyme activities and differences in antioxidant levels in spleen tissue. The entire procedure was carried out in an ice-bath environment. To minimize interference from residual blood, lipids, and other endogenous impurities within spleen tissue homogenates, a tissue-blank control was prepared for each sample: this control was prepared using the supernatant of the corresponding tissue homogenate without the addition of color-developing reagents, which was used to deduct background absorbance values caused by tissue impurities and eliminate systematic detection errors. During the detection of antioxidant indicators, the standardized operating procedures provided by the manufacturer were strictly followed to ensure data accuracy and reliability. Absorbance measurements and tissue protein concentrations were determined using a multi-functional microplate reader (Tiangen, Shanghai, China), and the corresponding antioxidant index results were finally calculated.

### 2.7. Gene Expression Analysis

Spleen tissue samples were removed from the liquid nitrogen tank. Twenty-milligram aliquots from each rabbit were weighed, minced with tissue scissors, and transferred to a centrifuge tube containing 1 mL TRIzol, mixed thoroughly by pipetting, and left at room temperature for 5 min to completely disrupt nucleoproteins and lyse cells; then 0.2 mL chloroform was added, the tube cap was tightly closed and vortexed vigorously for 15 s, left at room temperature for 2 min, and centrifuged at 4 °C, 12,000× *g* for 15 min to fully separate the organic and aqueous phases. The upper aqueous phase was carefully aspirated and transferred to a new centrifuge tube; 0.5 mL isopropanol was added and gently mixed by inversion, left at room temperature for 10 min, and centrifuged at 4 °C, 12,000× *g* for 10 min to pellet the RNA. The supernatant was discarded, 1 mL of 75% ethanol was added to gently wash the pellet, followed by centrifugation at 4 °C, 7500× *g* for 5 min; the supernatant was discarded, and the pellet was air-dried and dissolved in an appropriate amount of DEPC-treated water to obtain cellular mRNA. A reverse transcription reaction system was constructed, and a 20 μL reaction mixture containing the gDNA removal and reverse transcription reagents was prepared on ice, mixed by gentle pipetting, and briefly centrifuged to ensure even mixing. After incubation at 42 °C (15 min) and 95 °C (3 min), spleen cDNA was obtained. Finally, a 20 μL real-time PCR reaction mixture was prepared, and a qRT-PCR instrument (Singapore) was used to detect the expression levels of the following genes: heat shock protein genes—heat shock protein 90kDa alpha (cytosolic), class A member 1 (*HSP90AA1*), heat shock protein family A (Hsp70) member 4 (*HSPA4*), heat shock protein family D (*Hsp60*) member 1 (*HSPD1*); antioxidant genes—copper/zinc superoxide dismutase (*SOD1*), superoxide dismutase (*SOD2*), sirtuin 1 (*SIRT1*), sirtuin 2 (*SIRT2*), *GPX1*, *GPX3*; ferroptosis genes—*GPX4*, *NOX1*, *NOX2*, *NFE2L2*, *ACSL4*, *MAP1LC3A*, *PTGS2*, *LTF*, and *HMOX1*; cytokines—*IL1B*, *IL4*, and *TNF*. PCR primers were designed using the NCBI website (https://www.ncbi.nlm.nih.gov, accessed on 5 June 2024), and all primers were synthesized by Huada Gene Biotechnology Co., Ltd. (Shanghai, China). The primer sequences are listed in Table 1.

### 2.8. Enhanced Prussian Blue Staining: Detection of Fe Distribution in Tissues

Fe^3+^ in tissues reacts with potassium ferrocyanide to form blue ferrous ferrocyanide (Prussian blue) precipitate. Then, leveraging the peroxidase-like activity of Fe ions in the Prussian blue precipitate, addition of DAB and H_2_O_2_ catalyzes the oxidative polymerization of DAB to form a brown precipitate, thereby amplifying the signal and greatly enhancing the detection sensitivity for trace iron [52,53]. Paraffin blocks of spleen tissues from different groups were cut into 3–7 μm thick sections, floated on warm water to flatten, attached to glass slides, and baked at 60 °C for 2–3 h to ensure firm adhesion. The slides were placed in xylene for 15 min for dewaxing; then dehydrated with absolute ethanol and 75% ethanol sequentially, and washed with distilled water three times. Thereafter, the sections were placed in freshly prepared potassium ferrocyanide-hydrochloric acid staining solution, incubated at 37 °C for 20 min, and slowly rinsed with distilled water three times, 10 s each, until the rinse water was colorless. After DAB development, the sections were counterstained with hematoxylin for 4 min to stain nuclei blue-purple, and then rinsed with distilled water for 10 min. Subsequently, the sections were dehydrated with absolute ethanol for 5 min, cleared with xylene for 5 min, and an appropriate amount of neutral gum was applied for mounting; after the gum had naturally dried, iron distribution in different spleen tissues was observed under an optical microscope (Motic, AE31E, Xiamen, China) and photographed.

### 2.9. Immunohistochemical Staining of Spleen Tissue

Paraffin blocks of spleen tissues from different groups were processed as follows. Deparaffinization and rehydration: paraffin sections were sequentially placed in eco-friendly dewaxing solution I (Servicebio, Wuhan, China, Cat. No. G1128; main components: low-volatile isoparaffin mixtures, xylene-free) for 10 min → solution II (Servicebio, Cat. No. G1128, same formulation as Solution I) for 10 min → solution III (Servicebio, Cat. No. G1128, same formulation as Solution I) for 10 min → absolute ethanol I for 5 min → absolute ethanol II for 5 min → absolute ethanol III for 5 min → rinsed with distilled water. Antigen retrieval: after natural cooling, the slides were placed in PBS (pH 7.4) and washed on a rocking shaker three times, 5 min each. Endogenous peroxidase blocking: sections were placed in freshly prepared 3% H_2_O_2_-methanol solution, incubated at room temperature in the dark for 10 min, and then washed in PBS (pH 7.4) on a rocking shaker three times, 5 min each. Serum blocking: 3% BSA was added dropwise within the immunohistochemical circle to evenly cover the tissue, and the sections were blocked with BSA at room temperature for 30 min. The 3% BSA blocking solution reduces non-specific binding of primary or secondary antibodies to the slide, decreases background, and improves the signal-to-noise ratio for optimal staining. Primary antibody incubation: the blocking solution was gently shaken off, and primary antibodies (CD68 (Servicebio, Gb53109, 1:200), CD20 (Servicebio, Gb155721, 1:500), and CD3 (Servicebio, Gb13014, 1:500)) were added dropwise to the sections. The sections were placed flat in a humidified box and incubated overnight at 4 °C. Secondary antibody incubation: the slides were washed in PBS (pH 7.4) on a rocking shaker three times, 5 min each. After briefly drying the sections, HRP-labeled secondary antibody (Goat Anti-Rabbit IgG, Servicebio, catalog number GB23303, dilution 1:200) was added dropwise to cover the tissue and incubated at room temperature for 50 min. DAB development: the slides were washed in PBS (pH 7.4) on a rocking shaker three times, 5 min each. After briefly drying the sections, freshly prepared DAB developing solution was added dropwise within the circle, and development time was monitored under a microscope; positive staining appeared brownish-yellow, and the reaction was terminated by rinsing with tap water. Nuclear counterstaining: hematoxylin counterstaining for approximately 3 min → tap water wash → hematoxylin differentiation solution for a few seconds → tap water wash → hematoxylin bluing solution → running water wash. Dehydration and mounting: the sections were sequentially placed in 75% ethanol for 5 min → 85% ethanol for 5 min → absolute ethanol I for 5 min → absolute ethanol II for 5 min → n-butanol for 5 min → xylene I for 5 min for dehydration and clearing, then removed from xylene, slightly dried, and mounted with mounting medium. Microscopic examination: sections were observed under bright field using an inverted microscope (Motic, AE31E), and immunohistochemical images were captured.

### 2.10. Statistical Analysis

Data were first assessed using the Shapiro–Wilk normality test and Levene’s test for homogeneity of variances, followed by one-way analysis of variance (ANOVA) to compare differences between groups. If there is no “*”, it means *p* > 0.05; “*” means *p* < 0.05; “**” means *p* < 0.01; “***” means *p* < 0.001; “****” means *p* < 0.0001. The statistical results were completed by GraphPad Prism 9.0 software.

## 3. Results

### 3.1. Effects of Different Concentrations of Cr(VI) in Drinking Water on Spleen Coefficient of New Zealand Rabbits

**Figure 2:**
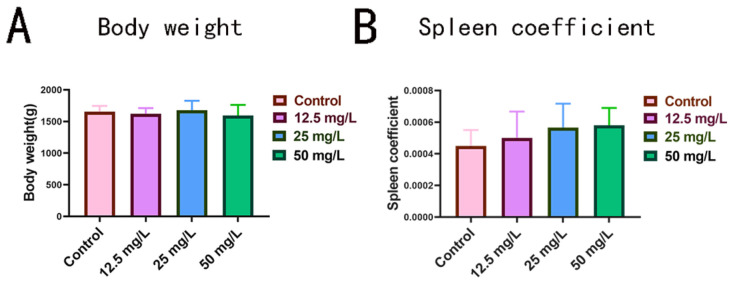
Effects of different concentrations of Cr(VI) on the spleen index of New Zealand rabbits. (**A**) Body weight after 28 days; (**B**) Spleen index after 28 days.

After four weeks of the experiment, the body weight of New Zealand rabbits was measured and recorded (Figure 2A). The mean body weights of the control, 12.5 mg/L, 25 mg/L, and 50 mg/L Cr(VI) groups were 1655.2 ± 90.07 g, 1623.3 ± 88.06 g, 1678.0 ± 148.90 g, and 1594.6 ± 168.51 g, respectively; no statistically significant differences were detected among the groups (*p* > 0.05). Similarly, the spleen coefficient did not differ significantly between the Cr(VI)-treated groups and the control group, as shown in Figure 2B (*p* > 0.05).

### 3.2. Pathological Damage of Spleen Tissue in New Zealand Rabbits Induced by Different Concentrations of Cr(VI) in Drinking Water

**Figure 3:**
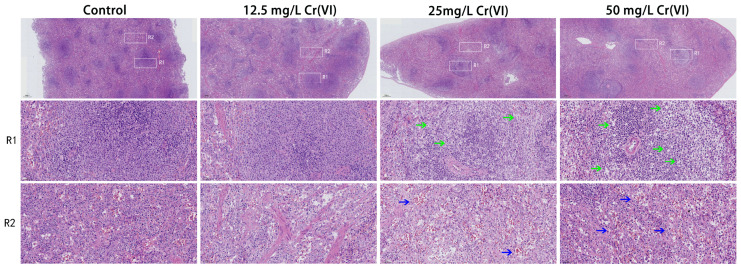
Effects of different concentrations of Cr(VI) on splenic histopathology in New Zealand rabbits (Scale bars: 200 μm and 30 μm). Green arrows in the figure mark atrophic splenocytes, while blue arrows denote dilated splenic sinusoids with increased erythrocytes inside the sinusoids. R1: White pulp area; R2: Red pulp area.

This study examined the pathological damage to spleen tissue following exposure to different Cr(VI) concentrations, focusing on the morphological and distributional changes in lymphocytes in the white pulp and red pulp. H&E staining results are shown in Figure 3. In the white pulp area of the control group, splenic lymphocytes in lymphoid follicles exhibited an intact structure, full morphology, and were densely and uniformly distributed. Compared with the control group, no obvious changes were observed in the 12.5 mg/L Cr(VI) group; in the 25 mg/L Cr(VI) group, splenic cells showed atrophy, and intercellular spaces were widened and appeared white (green arrows). The 50 mg/L Cr(VI) group exhibited the most severe damage, with numerous vacuolar structures between cells. Compared with the control group, lymphocytes in the white pulp lymphoid nodules after treatment with 12.5 mg/L and 25 mg/L Cr(VI) underwent atrophy and degeneration, characterized by lymphocyte shrinkage and widening of intercellular spaces. In the red pulp area, splenic sinuses in the control group were distributed as dot-like structures, with a small number of blood cells uniformly present. In the 12.5 mg/L Cr(VI) group, no obvious changes were noted. Compared with the control group, in the 25 mg/L and 50 mg/L Cr(VI) groups, splenic sinuses were enlarged and elongated, and the number of red blood cells was increased (blue arrows). Our results indicate that Cr(VI)-induced damage to splenic lymphocyte distribution is concentration-dependent and causes atrophy, degeneration, and increased hemorrhage in spleen tissues.

### 3.3. Effects of Different Concentrations of Cr(VI) on the Distribution of Immune Cells and the Expression of Inflammatory Factor-Related Genes in Spleen Tissue of New Zealand Rabbits

**Figure 4:**
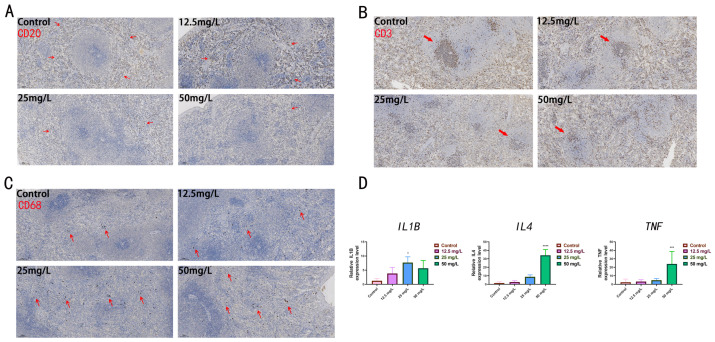
Effects of different Cr(VI) concentrations on immune cell distribution in spleen tissue sections and inflammatory factor gene expression in spleen tissues. (**A**) B-cell distribution in spleen sections. Red arrows indicate B-cells. (**B**) T-cell distribution in spleen sections. Red arrows indicate T-cells. (**C**) Macrophage distribution in spleen sections. Red arrows indicate macrophages. (**D**) Inflammatory factor gene expression in spleen tissues by qRT-PCR. *: *p* < 0.05, ***: *p* < 0.001, ****: *p* < 0.0001 vs. control.

The effects of Cr(VI) on the distribution of major immune cells and the expression of immune factor-related genes in spleen tissue are shown in Figure 4. Immunohistochemistry revealed that with increasing Cr(VI) concentration, the expression rates of CD20 and CD3 antigens were down-regulated, indicating reduced splenic B cell and T cell levels (Figure 4A,B); conversely, the expression rate of CD68 increased (Figure 4C). At 12.5 mg/L Cr(VI), B cell distribution was similar to that in the control group; at 25 mg/L, a modest decrease in B-cell numbers was observed; at 50 mg/L, B-cell distribution became more sparse (Figure 4A). In the control group, numerous CD3-positive T lymphocytes were distributed around the PALS area. At 12.5 mg/L Cr(VI), T-cell distribution was similar to control; at 25 mg/L, T-cell distribution around the PALS decreased modestly; at 50 mg/L, T cell distribution became more sparse (Figure 4B). Macrophage distribution at 12.5 mg/L was similar to control; at 25 mg/L, a modest increase in macrophages around splenic blood vessels was noted; at 50 mg/L, more macrophages were observed around blood vessels (Figure 4C). Cr(VI) treatment altered the mRNA expression of *IL1B*, *IL4*, and *TNF* compared with the control group. Compared with the control group, 12.5 mg/L Cr(VI) showed no obvious effect on *IL1B*, *IL4*, or *TNF* expression (*p* > 0.05). In contrast, 12.5 mg/L Cr(VI) significantly upregulated *IL1B* expression (*p* < 0.05) without affecting *IL4* or *TNF* (*p* > 0.05). Furthermore, 50 mg/L Cr(VI) significantly increased *IL4* (*p* < 0.0001) and *TNF* (*p* < 0.001) expression, but did not significantly alter *IL1B* expression (*p* > 0.05) (Figure 4D). These results indicate that Cr(VI) down-regulates splenic immune cell levels and induces increased expression of splenic inflammatory factor genes.

### 3.4. Effects of Different Concentrations of Cr(VI) on Antioxidant Capacity of Spleen Tissue in New Zealand Rabbits

**Figure 5:**
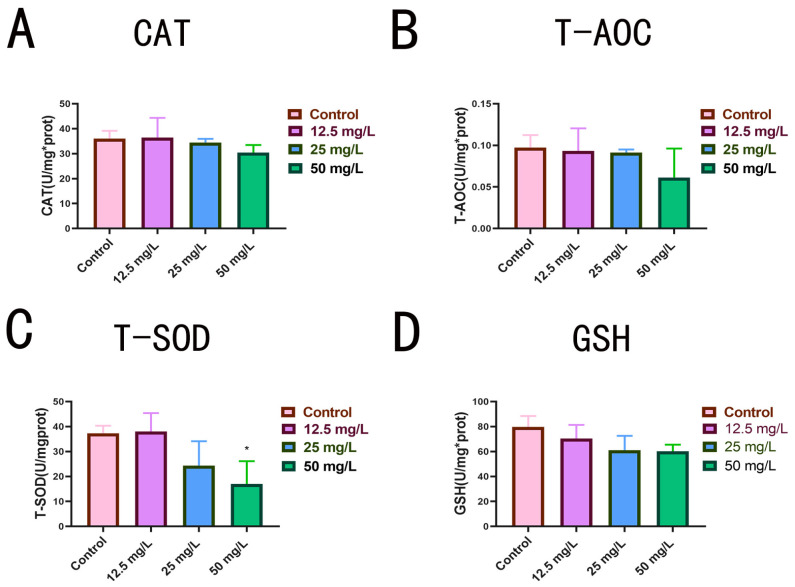
Effects of different Cr(VI) concentrations on the T-AOC level, GSH content, CAT and T-SOD activities in spleen homogenates. (**A**) The CAT activities of spleen homogenates; (**B**) The T-AOC level of spleen homogenates; (**C**) The T-SOD activities of spleen homogenates; (**D**) The GSH level of spleen homogenates. *: *p* < 0.05 vs. control.

The effects of Cr(VI) on enzyme activity parameters in spleen tissue are shown in Figure 5. Compared with the control group, at 12.5 mg/L Cr(VI), CAT activity, T-SOD activity, T-AOC level, and GSH level did not differ significantly from the control group (*p* > 0.05; Figure 5). At 25 mg/L Cr(VI), no statistically significant differences were observed in T-AOC, GSH, T-SOD, or CAT levels compared with the control group (*p* > 0.05). At 50 mg/L Cr(VI), T-SOD activity was significantly decreased (*p* < 0.05), whereas T-AOC level, GSH level, and CAT activity did not differ significantly from the control (*p* > 0.05). These results indicate that Cr(VI) induces down-regulation of the splenic antioxidant capacity in New Zealand rabbits, leading to consumption of antioxidant substances.

### 3.5. Effects of Different Concentrations of Cr(VI) on the Expression of Antioxidant-Related Genes in Spleen Tissue of New Zealand Rabbits

**Figure 6:**
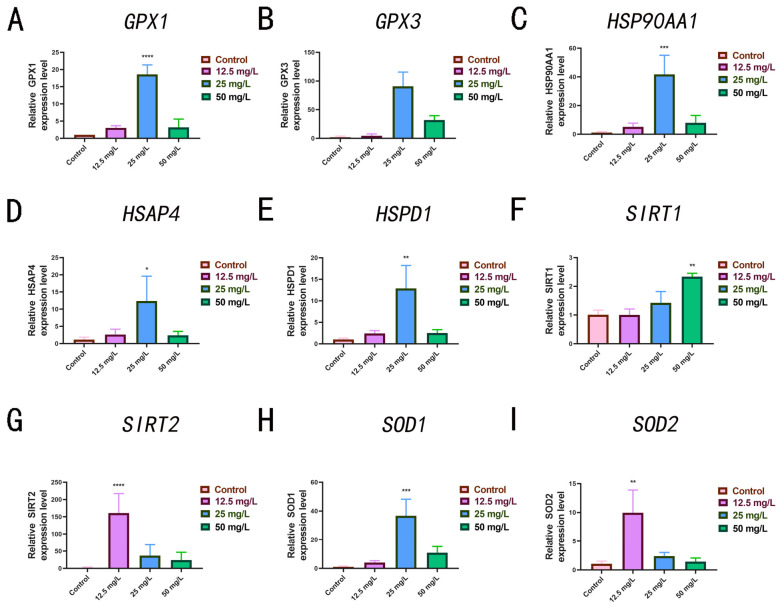
Effects of different Cr(VI) concentrations on antioxidant-related gene expression in spleen tissue. (**A**–**I**) qRT-PCR analysis of *HSP90AA1*, *HSPA4*, *HSPD1*, *SOD1*, *SOD2*, *GPX1*, *GPX3*, *SIRT1*, *and SIRT2* mRNA levels in spleen tissue, respectively. *: *p* < 0.05, **: *p* < 0.01, ***: *p* < 0.001, ****: *p* < 0.0001 vs. control.

The potent oxidizing property of Cr(VI) is the primary driver of its biological toxicity. Therefore, qRT-PCR was used to assess the expression of antioxidant-related genes (Figure 6). At 12.5 mg/L Cr(VI), only *SIRT2* and *SOD2* showed statistically significant upregulation compared with the control group (*p* < 0.0001, *p* < 0.01), while no significant differences were observed for the other genes (*p* > 0.05). At 25 mg/L Cr(VI), significant upregulation was detected for *HSP90AA1*, *HSPA4*, *HSPD1*, *SOD1*, *GPX1*, and *GPX3* (*p* < 0.001, *p* < 0.05, *p* < 0.01 and *p* < 0.001), whereas the expression changes in *SIRT1*, *SIRT2*, and *SOD2* did not reach statistical significance (*p* > 0.05). Under the 50 mg/L Cr(VI) condition, only the expression level of *SIRT1* was significantly upregulated compared with the control group (*p* < 0.01), while no statistically significant differences were observed for the other genes. These results indicate that different Cr(VI) concentrations induce an antioxidant defense response in spleen tissue.

### 3.6. Effects of Different Concentrations of Cr(VI) on Iron Element Distribution in Spleen Tissue of New Zealand Rabbits

**Figure 7:**
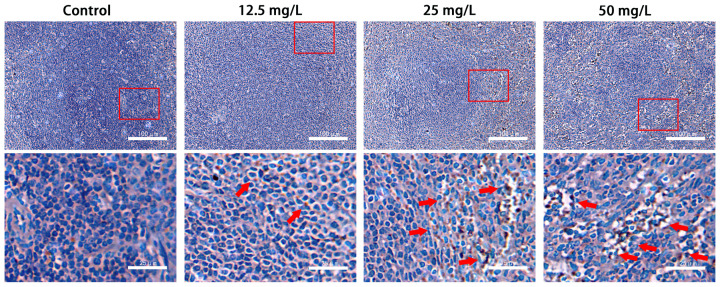
Enhanced Prussian blue staining of spleen tissue (scale bar: 100 μm and 25 μm). The area enclosed by the red box in the upper figure corresponds to the magnified region shown in the lower-panel image. Red arrows indicate Fe^3+^-positive brownish-yellow staining.

A significant marker of ferroptosis in animal tissues is the accumulation of Fe elements in cells. This study used enhanced Prussian blue staining technology to detect the content of iron (Fe^3+^) in spleen tissue. The histopathological observation results of enhanced Prussian blue staining sections of spleen tissue of New Zealand rabbits are shown in Figure 7. The spleen tissue sections of the Control group showed that the nucleus was dark blue and full in morphology, and there were few positive brown areas in tissue cells. With the increase in Cr(VI) concentration, under the conditions of 12.5 mg/L Cr(VI), 25 mg/L Cr(VI) and 50 mg/L Cr(VI), the brown Fe deposition positive areas between tissue cells gradually increased. These results confirm at the histological level that Cr(VI) exposure leads to iron ion accumulation in the spleen, providing morphological evidence for the occurrence of ferroptosis.

### 3.7. Effects of Different Concentrations of Cr(VI) on the Expression of Ferroptosis-Related Genes in Spleen Tissue of New Zealand Rabbits

The aforementioned experiments confirmed iron deposition in the spleen tissue of New Zealand rabbits at the histological level. Subsequently, qRT-PCR was used to detect the expression of ferroptosis-related genes. The results were as follows: Regarding *ACSL4*, no significant difference was observed at 12.5 mg/L Cr(VI) compared with the control group, while a significant upregulation was detected at 25 mg/L and 50 mg/L Cr(VI) (*p* < 0.001). Compared with the control group, no statistically significant differences in *ALOX15* expression were found at 12.5 mg/L or 25 mg/L Cr(VI), whereas a significant downregulation was observed at 50 mg/L Cr(VI) (*p* < 0.05). The expression of *NOX1* increased in a dose-dependent manner, though statistical significance was not always reached. *NOX2* expression was significantly upregulated at 25 mg/L (*p* < 0.001) and 50 mg/L Cr(VI) (*p* < 0.05) compared to the control; the difference at 12.5 mg/L was not statistically significant. For *NOX4*, no significant differences were found between any Cr(VI) treatment group and the control group. *HMOX1* expression was significantly higher than the control at all Cr(VI) concentrations (*p* < 0.001). For *GPX4*, a significant upregulation was observed only at 25 mg/L Cr(VI) (*p* < 0.05), with no significant differences from the control at 12.5 mg/L or 50 mg/L. No statistically significant differences in *NFE2L2* expression were found between any treatment group and the control. *PTGS2* expression was significantly upregulated at 50 mg/L Cr(VI) (*p* < 0.05), while the change at 25 mg/L was not statistically significant. *MAP1LC3A* gene expression showed no significant difference from the control at any concentration. *LTF* gene expression did not differ significantly from the control at any Cr(VI) concentration. *MTF1* gene expression was significantly upregulated only at 50 mg/L Cr(VI) (*p* < 0.001). These results indicate that high concentrations of Cr(VI) can upregulate the expression of certain pro-ferroptosis genes and downregulate some anti-ferroptosis genes, which may contribute to the induction of ferroptosis in spleen tissue.

## 4. Discussion

Ferroptosis, an Fe-dependent, lipid peroxidation-driven regulated cell death, has garnered increasing attention in heavy metal-induced immunotoxicity [54,55]. This study provides the first evidence in the spleen of New Zealand rabbits that Cr(VI) exposure induces ferroptosis in splenic lymphocytes by depleting GSH, downregulating *GPX4* expression, and activating the ACSL4-NOX-mediated lipid peroxidation positive feedback loop at the transcriptional level [56]. A key mechanistic insight of this study is that Cr(VI) induces immune injury by disrupting the interplay between splenic redox balance, lipid peroxidation, and iron metabolism, thereby linking oxidative stress, ferroptosis, and immune injury at the metabolic level and elucidating the metabolic basis of Cr(VI)-induced splenic immunotoxicity.

Cr(VI) exposure in this study exhibited a clear concentration-dependent toxic effect. Although no statistically significant difference in body weight was observed among groups (Figure 2A), a trend toward weight reduction was noted in the 50 mg/L group, suggesting an overall catabolic state under high-dose Cr(VI) stress. This finding suggests that under low-level Cr(VI) exposure, New Zealand rabbits may maintain homeostasis by activating Nrf2-mediated antioxidant defense and the reductive detoxification of Cr(VI) to Cr(III); however, when the exposure dose exceeds the body’s compensatory capacity (≥50 mg/L), the detoxification system becomes saturated and toxic effects emerge. The slight weight increase in the 25 mg/L group may reflect a metabolic adaptive response to low-dose stress, though the underlying mechanism requires further validation by metabolomics and energy metabolism analyses. The increase in spleen organ index may be related to the hemorrhage and edema observed in pathological sections (Figure 3) and may also be influenced by changes in body weight.

The structural integrity of splenic white pulp is fundamental to immune function, with T cells enriched in the PALS, B cells in lymphoid follicles, and macrophages in the marginal zone. CD20 is a surface marker on B cells, expressed from pre-B to mature B cells, and plays important roles in B-cell development, differentiation, and signal transduction [57]. CD3 is a surface marker on T cells, expressed on almost all mature T cells, with T cells mainly localized around splenic blood vessels [58]. CD3 forms a complex with the T-cell receptor, participating in T-cell activation and signal transduction, and thus plays a key role in the immune response. CD68 is a macrophage marker, expressed mainly in macrophages, dendritic cells, and monocytes, and is frequently used to study phagocytic function and inflammatory responses [59]. Our study shows that Cr(VI) reduces the percentages of CD20-positive and CD3-positive cells in spleen tissue, while the percentage of CD68-positive cells increases with increasing concentration, the latter indicating an inflammatory reaction in the spleen. Furthermore, as a key immune organ, the spleen is the central site for immune cell aggregation and initiation of immune responses, with cytokines playing a pivotal role in maintaining and regulating immune function. Oxidative stress can trigger the release of inflammatory cytokines and induce inflammatory reactions; among these, interleukins and TNF mediate local and systemic inflammation, and the degree of oxidative stress damage can be assessed by measuring cytokine gene expression [60,61]. The IL1β protein encoded by the *IL1B* gene is a pro-inflammatory cytokine involved in inflammatory and immune responses [62]. The IL4 protein encoded by the *IL4* gene is an anti-inflammatory cytokine that plays an important role in splenic immune regulation, allergic reactions, and tissue repair [63,64]. TNF is a small protein secreted by macrophages and is also a multifunctional pro-inflammatory cytokine [65]. Irfan Rahman demonstrated in a study of oxidative stress, transcription factors, and chromatin remodeling in lung inflammation that under oxidative stress, transcription factors such as AP-1 and NF-κB are activated and bind to the promoter region of the *TNF* gene, promoting its transcription and thereby increasing *TNF* expression and release [66]. In this study, compared with the control group, the expression levels of *IL1B*, *IL4*, and *TNF* were progressively increased with increasing Cr(VI) concentration, indicating that Cr(VI) exposure induces oxidative stress, leading to the release of inflammatory factors and the promotion of inflammatory reactions in splenic cells. Moreover, the observed decrease in B cells and T cells suggests that splenic lymphocytes may be the primary target cells of Cr(VI)-induced ferroptosis. Notably, the decrease in T cells in the PALS area may be related to the relatively low GSH reserve in this region. The concentration-dependent increase in macrophage numbers may reflect involvement of macrophages in two pathological processes: phagocytosis of ferroptotic debris and inflammatory activation, with release of pro-inflammatory factors that further amplify oxidative stress. The up-regulation of inflammatory factors such as IL1β and TNF may exacerbate ferroptosis via a positive feedback loop, forming an “oxidative stress–inflammation–ferroptosis” vicious cycle. Consistent with the hematological results from the same batch of animals showing that Cr(VI) reduces various peripheral blood immune cells in New Zealand rabbits, our findings of decreased splenic immune cell content are highly congruent [67]. Splenic T and B cells are depleted by ferroptosis, resulting in an insufficient supply of peripheral lymphocytes; activated macrophages and inflammatory factors can clear circulating blood cells and inhibit hematopoiesis, jointly mediating the reduction in peripheral blood cell counts. These pathological processes all stem from disturbed splenic metabolic homeostasis, strongly confirming that immune metabolic dysregulation is the core driver of Cr(VI)-induced splenic immune injury.

The strong oxidizing property of Cr(VI) is the core driving force of its toxicity, and defense against oxidative stress is a critical aspect of cellular function, involving a variety of antioxidant genes. The antioxidant defense system includes SOD and GPX family enzymes, which catalyze the dismutation of superoxide radicals and a reduction in peroxides, respectively [68]. SIRT1 and SIRT2, as NAD^+^-dependent deacetylases, can enhance antioxidant enzyme activity via FoxO transcription factors, thereby reducing ROS accumulation [69]. Heat shock proteins (HSPs) maintain protein stability and enhance stress resistance. Among them, HSP90α, a potential tumor marker [70,71], is upregulated through the NF-κB pathway under oxidative stress, assisting in protein folding and promoting antioxidant enzyme production [72]. The mitochondrial chaperone HSP60/HSP10 complex (encoded by HSPD1/HSPE1) refolds oxidatively damaged proteins, mitigating cellular damage [73], while HSPA4 plays a role in intracellular protein transport and stress responses [74]. Consistent with these protective roles, upregulation of multiple *HSP* genes, including *Hsp90aa1*, *Hspa1a*, *Hspa4*, and *Hspa5*, has been observed in oxidative stress models [75]. In this study, splenic T-AOC, T-SOD activity, and GSH content showed a downward trend with increasing Cr(VI) exposure concentration, whereas antioxidant genes (*SOD1*, *SOD2*, *GPX1*, *GPX3*) and heat shock protein genes (*HSP90AA1*, *HSPA4*, *HSPD1*) were up-regulated in the low- and medium-dose groups (12.5–25 mg/L Cr(VI)) and decreased in the high-dose group (50 mg/L Cr(VI)) but remained higher than those in the control group. This finding suggests that low and medium dose Cr(VI) exposure activates the oxidative defense of splenic cells, while high-dose Cr(VI) exposure leads to exhaustion of the antioxidant system and attenuation of compensatory capacity. The persistent up-regulation of *SIRT1* and *SIRT2* may represent a delayed protective mechanism, but whether their deacetylase activity is sufficient to reverse oxidative damage in the high-dose group requires functional validation. This study shows that Cr(VI) can increase the expression levels of antioxidant-related genes including *GPX*s, *SIRT*s, and *SOD*s in spleen tissue, which likely represents a defensive response to Cr(VI) exposure.

**Figure 8:**
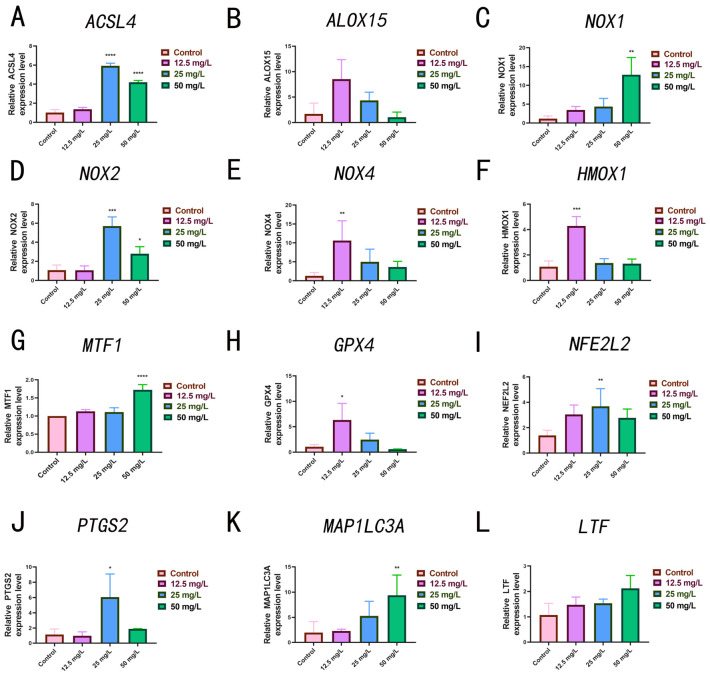
(**A**–**L**): Effects of different concentrations of Cr(VI) supplemented in drinking water on the expression of ferroptosis-related genes in the spleen tissue of New Zealand rabbits. *: *p* < 0.05, **: *p* < 0.01, ***: *p* < 0.001, ****: *p* < 0.0001 vs. control.

Based on the observed changes in ferroptosis-related markers, this study demonstrates that exposure to different Cr(VI) concentrations induces three distinct degrees of splenic ferroptosis injury in New Zealand rabbits. First, after 4 weeks of 12.5 mg/L Cr(VI), *GPX4* was compensatorily up-regulated, forming a synergistic antioxidant network with *GPX1* and *GPX3*, and the peak expression of *HMOX1* indicated activation of the heme degradation pathway. Second, after 4 weeks of 25 mg/L Cr(VI), GSH levels decreased, but high *GPX4* expression maintained compensation, and the ACSL4-NOX2 axis was activated, initiating a lipid peroxidation positive feedback loop. Third, after 4 weeks of 50 mg/L Cr(VI), *GPX4* expression was down-regulated, the antioxidant defense collapsed, and ferroptosis entered an active stage. ACSL4 increases lipid peroxidation substrates by esterifying long-chain PUFAs into membrane phospholipids; ALOX15 directly catalyzes PUFA peroxidation, forming a “substrate supply–peroxidation execution” positive feedback loop with ACSL4; NOX1/2 produce O_2_^−^, providing an oxidative stress foundation for lipid peroxidation. At 50 mg/L Cr(VI), the down-regulation of *GPX4* marks the collapse of GPX4-mediated antioxidant defense. Additionally, iron deposition in the splenic red pulp area not only reflects ferroptosis but may also contribute to sinus dilation and impaired blood filtration, which is consistent with the pathological observation of “splenic sinus enlargement and increased red blood cells”. The down-regulation of *ALOX15* in the 50 mg/L Cr(VI) group does not indicate inactivation of lipid peroxidation but rather reflects exhaustion under severe and sustained oxidative stress. At this high dose, lipid peroxidation is driven mainly by ACSL4-mediated substrate supply and NOX-generated ROS, which together maintain the positive feedback loop and trigger overt ferroptosis. Although GPX1, GPX3, and GPX4 belong to the same GPX family, they have different effects on ferroptosis. GPX1 primarily reduces lipid peroxidation by clearing H_2_O_2_ and inhibits ferroptosis [76]; GPX3 inhibits ferroptosis by regulating the Nrf2/GPX4 pathway and maintaining redox balance [77]. These two enzymes play important antioxidant and anti-ferroptotic roles under different physiological and pathological conditions. Furthermore, up-regulation of the pro-ferroptosis genes *NOX1*, *NOX2*, *NOX4*, *ACSL4*, *PTGS2*, and *HMOX1* promotes ferroptosis. In this study, compared with the control group, *GPX4* expression first increased and then decreased, indicating that GPX4 plays an antioxidant role at 12.5 and 25 mg/L Cr(VI), while at 50 mg/L Cr(VI), GPX4 fails to effectively detoxify lipid peroxides, leading to ferroptosis. The expression levels of *NOX1*, *NOX2*, *NOX4*, and *ACSL4* all showed an upward trend, confirming that ferroptosis occurred in the tested tissue. NOX enzymes constitute a multi-gene family and are the only enzymes that directly produce ROS. As members of the NOX family, NOX1 and NOX2 reduce O_2_ to O_2_^−^ through an NADPH-dependent single-electron reduction process, representing a major source of ROS in vivo, and excessive ROS attack polyunsaturated fatty acids on the cell membrane, triggering lipid peroxidation and leading to ferroptosis [78]. In contrast to the significant upregulation of *NOX1* and *NOX2*, the expression of *NOX4* remained unchanged across all Cr(VI) treatment groups (Figure 8). This differential response among NOX isoforms is noteworthy and may be attributed to their distinct regulatory mechanisms. Unlike NOX1 and NOX2, which are acutely activated by stress signaling and primarily produce superoxide (O_2_^−^), NOX4 is constitutively active and predominantly generates hydrogen peroxide (H_2_O_2_) [79]. The lack of NOX4 induction in the present study suggests that Cr(VI)-induced splenic ferroptosis is driven mainly by an acute superoxide burst via NOX1/NOX2 activation, rather than sustained H_2_O_2_ production through NOX4. This isoform-specific pattern reinforces the concept that distinct NOX family members play non-redundant roles in redox-dependent cell death pathways, and further supports the conclusion that the ACSL4–NOX1/2 lipid peroxidation axis, rather than NOX4, is the key mediator of ferroptosis in Cr(VI)-exposed rabbit spleen. ACSL4 plays a key role in regulating lipid metabolism by esterifying PUFAs and incorporating them into membrane phospholipids, thereby accelerating the rate of lipid peroxidation and promoting ferroptosis [80]. As a key enzyme regulating heme metabolism, HO-1 catalyzes the degradation of heme to release Fe^2+^, thereby promoting substantial iron accumulation in cells, leading to iron dyshomeostasis and further promoting ferroptosis [81,82,83]. PTGS2 is a key enzyme in the initial step of prostaglandin synthesis and can regulate ferroptosis sensitivity by modulating the levels of key membrane phospholipids [84]. From the perspective of the complex regulatory network of ferroptosis, the System Xc^−^/GSH/GPX4 antioxidant axis and the ACSL4/ALOX15 lipid peroxidation axis represent two counterbalancing forces. This study reveals that under Cr(VI) exposure, activation of the Nrf2-GPX4 pathway in rabbit spleen tissue is dose-dependent. Low-dose Cr(VI) successfully activates this protective pathway, while high-dose Cr(VI) disrupts the balance and triggers ferroptosis. The role of the p53-SLC7A11 axis in this process and the specific involvement of other transcription factors such as ATF4 require further investigation.

Finally, although this study systematically elucidates the mechanism of Cr(VI)-induced splenic ferroptosis via oxidative stress pathways, several limitations should be acknowledged. First, this study primarily relied on gene expression analysis and did not include direct evidence of ferroptosis at the protein level, such as GPX4 protein or lipid peroxidation products (e.g., malondialdehyde, 4-hydroxynonenal). Second, although iron accumulation was observed by Prussian blue staining, we did not quantify iron content or the expression of iron metabolism-related proteins (e.g., ferritin, transferrin receptor). Third, this study did not utilize specific ferroptosis inhibitors (e.g., ferrostatin-1, liproxstatin-1) to confirm the causal relationship between ferroptosis and the observed immune injury. Fourth, the 4-week exposure period used in this study corresponds to an acute/subacute toxicity model, whereas real-world human and animal exposure to Cr(VI) typically occurs over years. This short duration does not allow us to rule out compensatory responses or delayed pathological effects. Therefore, our conclusions should be considered preliminary and require validation in long-term exposure models. Fifth, the thioredoxin and glutaredoxin antioxidant systems, particularly the mitochondrial Trx2/Prx3 axis, were not examined and warrant dedicated investigation in future studies [85]. Sixth, although each group contained both male and female rabbits, sex-specific analyses were not performed; males and females were analyzed together, which may have masked potential sex-dependent differences in immune and oxidative stress responses. Future studies should address these gaps at both the protein and functional levels to achieve a more comprehensive understanding of the metabolic regulatory network underlying Cr(VI)-induced splenic immunotoxicity.

## 5. Conclusions

In summary, this study reveals for the first time that ferroptosis is a key novel mechanism underlying Cr(VI)-induced spleen injury in rabbits, characterized by a malignant coupling of iron metabolism disturbance and antioxidant system dysregulation. This finding not only enriches the understanding of the molecular basis of Cr(VI) immunotoxicity but also provides new insights into targeting key ferroptosis-related genes as potential biomarkers and intervention targets for immune damage caused by environmental Cr(VI) exposure.

## Figures and Tables

**Table 1:** Primer sequences of the detected genes.

Gene	Forward Primer	Reverse Primer
*HSP90AA1*	GCCCAGAGTGCTGAATACCC	TAACAGGTGCCCTGCTTCTC
*HSPA4*	TCCAGTGCCTCTCTAGTGGA	AGCTTGAGAAGTCACGCCG
*HSPD1*	GTAAGCCCCTGGTCATAATTGCT	TCCCTCTTCTCCAAATACTGCAC
*SOD1*	CACCATCCACTTCGAGCAGA	CTGCACTCGTACAGCCTTGT
*SOD2*	GCAAGGAACAACAGGCCTTA	AACAGCCAGAGAGCACGAC
*GPX1*	AACCAGTTTGGGCATCAGGAGA	GGCCTTGGCGCCGTT
*GPX3*	GGGGCCAAGAGAAGTCCAA	TCTTGTAGTGCATTCAGTTCCACG
*GPX4*	CGGGAGGCAGGAGCC	GGTGAAGTTCCACTTGATGGC
*IL1B*	AACCTCTTCGAGGCACAAGG	GTCCTGGAAGGAGCACTTCAT
*IL4*	CTCACAGAGAGAAAGAACACAAC	TCCTTCCCAGGACAGTTGC
*TNF*	ATGGTCACCCTCAGATCAGC	GTTGTCCGTGAGCTTCATGC
*NOX1*	CAGCTCCTGACGATGGGAAA	GCCAATGCAGACCCAAGGA
*NOX2*	TGATCCAGTGATGTGTGAGCAA	AGGGTGAGTGACCACCTTGG
*NOX4*	GGATCCCAGAAGGTCCCAAG	CATTGTAGTTCACTGAGAAGTTGAG
*ALOX15*	GGTTCACGTGGGTCCCTAAC	TGGCCCAAAGGCACCATAAT
*PTGS2*	GTCAAAACCGAGGTGTGTGC	TCAGAAATTCCGGCGTGGAG
*LTF*	TCTATTTCGACCGCGACGAT	TCCTGGGACGGCTTCTGC
*ACSL4*	GGCCCCACTGTCCCC	TTGTATAACCGCCTTCCTGC
*HMOX1*	GCCGAGGGTTTTAAGCTGGT	CAGCTCCTCCGGGAAGTAGA
*ACTB*	CAGTGGCCGTACAACTGGTAT	AAACGCAAGATCGCATGTGG

## Data Availability

Data can be obtained from the first author upon reasonable request.

## Abbreviations

The following abbreviations are used in this manuscript:

Cr	Chromium
Cr(III)	Trivalent chromium
Cr(VI)	Hexavalent chromium
ROS	Reactive oxygen species
PALS	periarteriolar lymphoid sheath
WP	white pulp
RP	red pulp
H&E	hematoxylin-eosin
MZ	marginal zone
NCBI	National Center for Biotechnology Information
IVC	individually ventilated cage
SOD1	Superoxide dismutase 1
GPX	Glutathione peroxidase
HSPs	Heat shock proteins
PTGS2	Prostaglandin-endoperoxide synthase 2
ACSL4	Acyl-CoA Synthetase Long Chain Family Member 4
ALOX15	Arachidonate 15-Lipoxygenase
HMOX1	Heme Oxygenase 1
LTF	Lactoferrin
MTF1	Metal Regulatory Transcription Factor 1
NOX1	NADPH Oxidase 1
NOX2	NADPH Oxidase 2
NOX4	NADPH Oxidase 4
HSP90AA1	Heat Shock Protein 90kDa Alpha (Cytosolic), Class A Member 1
HSPA4	Heat Shock Protein Family A (Hsp70) Member 4
HSPD1	Heat Shock Protein Family D (Hsp60) Member 1
PUFAs	Polyunsaturated fatty acids
